# Primary Central Nervous System Lymphoma: A Case of Solitary Brain Lesion

**DOI:** 10.7759/cureus.15966

**Published:** 2021-06-27

**Authors:** Halimat Lawal, Nya Anwanane, Anas Atrash

**Affiliations:** 1 Internal Medicine, University of Pittsburgh Medical Center Pinnacle, Harrisburg, USA; 2 Internal Medicine, Oakland University, Michigan, USA

**Keywords:** primary cns lymphoma, solitary brain lesions, high grade lymphoma, lymphoma, cns lymphoma, brain tumors, b cell lymphoma, extra nodal non hodgkin lymphoma, lymphoma versus glioma

## Abstract

Primary central nervous system lymphoma (PCNSL) is an uncommon variant of extranodal non-Hodgkin’s lymphoma (NHL) that involves the brain, leptomeninges, eyes, or spinal cord without evidence of systemic disease.

This article presents a case of a 63-year-old Caucasian female with high-grade B-cell lymphoma who was found to have a solitary brain lesion and no distant metastasis upon diagnosis. The patient had left-sided weakness and difficulty standing on presentation and was found to have a right frontal lobe mass with surrounding mild vasogenic edema and a small focal area of hemorrhage concerning for high-grade glioma versus lymphoma on MRI. However, pathology results revealed high-grade B-cell lymphoma.

The case reinforces the importance of working up any lesion suspicious for lymphoma/glioma. Diagnosis of high-grade B-cell lymphoma can be difficult based on morphological and cytological appearance due to varying gene expression and presentation at diagnosis. It can closely mimic diffuse B-cell lymphoma. Extensive workup including HIV serology, MRI imaging, evaluation for spinal cord involvement, and lumbar puncture (LP), to rule out cerebrospinal fluid (CSF) involvement, prior to initiating treatment needs to be done. The case also addresses high-grade methotrexate (MTX)-based chemotherapy as a treatment that improves mortality in patients with primary central nervous system (CNS) lymphoma.

## Introduction

Primary central nervous system lymphoma (PCNSL) is an uncommon variant of extranodal non-Hodgkin’s lymphoma (NHL) that involves the brain, leptomeninges, eyes, or spinal cord without evidence of systemic disease. Here we present a case of a 63-year-old Caucasian female with high-grade B-cell lymphoma who was found to have a solitary brain lesion and no distant metastasis upon diagnosis.

## Case presentation

**Figure 1:**
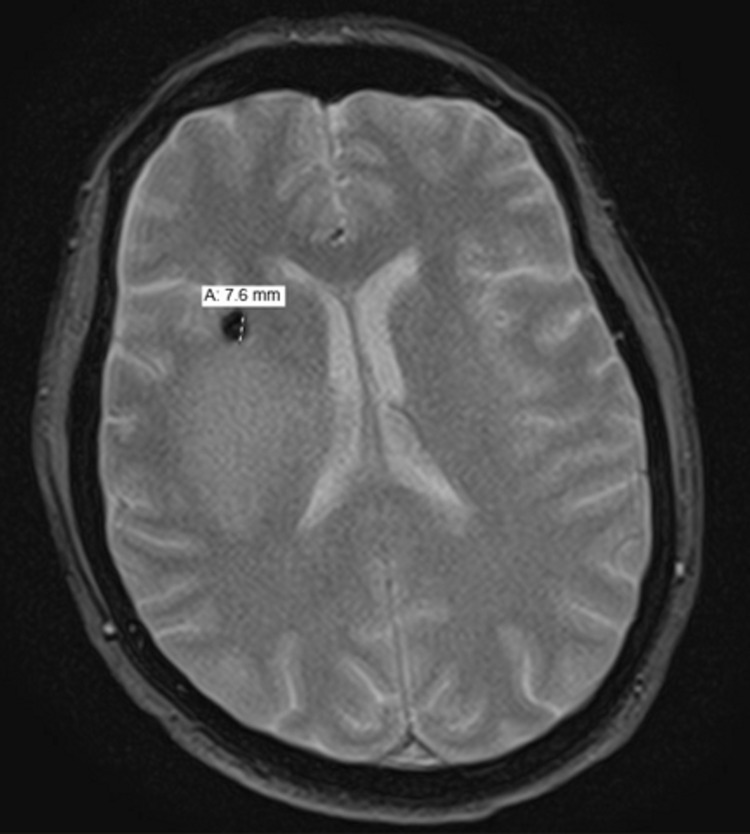
Pre-op MRI showing mass with surrounding vasogenic edema.

A 63-year-old Caucasian female with a past medical history (PMH) of arthritis presented originally to the hospital due to an abnormal MRI. The patient was having weakness on the left side for the past few weeks along with difficulty concentrating. Her symptoms started to worsen when she was leaning towards the left upon standing, veering to the left when driving, and had difficulty standing from a seated position. She was evaluated by her primary care physician (PCP) who ordered a brain MRI. The MRI showed a right frontal lobe mass with surrounding mild vasogenic edema and a small focal area of hemorrhage as seen in Figure 1.

**Figure 2:**
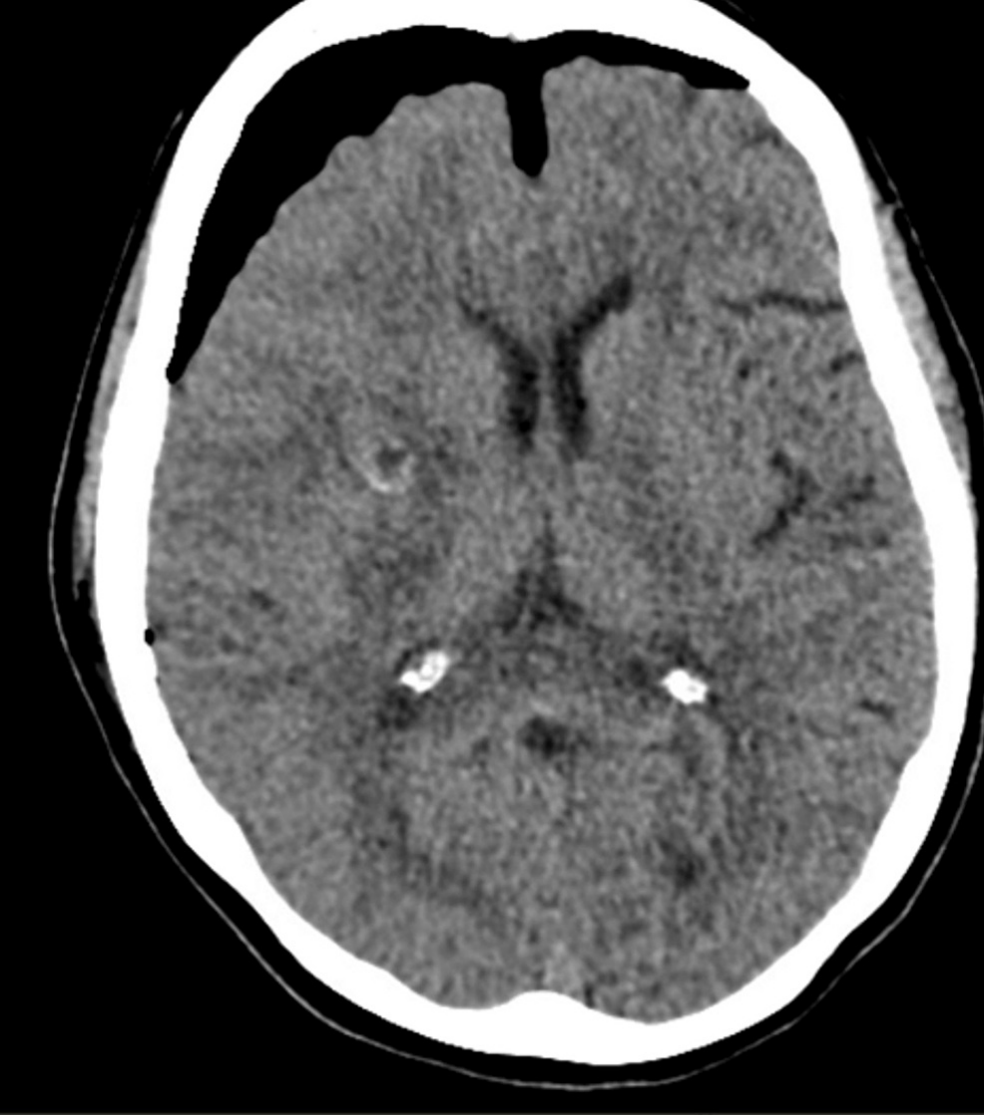
Post-op CT scan showing post-surgical changes and residual acute blood.

These findings were concerning for high-grade glioma with other differentials including lymphoma and other causes. She was then sent to the hospital for further evaluation. Family history is significant for NHL in the father and brother diagnosed with NHL ~15years ago, who are in remission. On initial exam, vitals were significant for mildly elevated blood pressure but otherwise unremarkable. On neurologic exam, the patient was notably alert and oriented x 3, with intact speech but mild difficulty swallowing. She had mild left pronator drift, left-sided facial droop, left hemiparesis with 4/5 strength on the left upper and lower extremity, and 5/5 strength on the right upper and lower extremity. Sensations were intact in all extremities. She was started on 500 mg of Keppra, every 12 h and 4 mg of dexamethasone, every 8 h. A CT chest/abdomen/pelvis was obtained which showed no evidence of metastatic disease. Neurosurgery evaluated the patient and she underwent right frontoparietal craniotomy and partial removal of the tumor. The patient had a post-surgical brain CT that showed small amounts of high attenuation material in gas bubbles along the track of the resection, likely representing residual acute blood (Figure 2).

**Figure 3:**
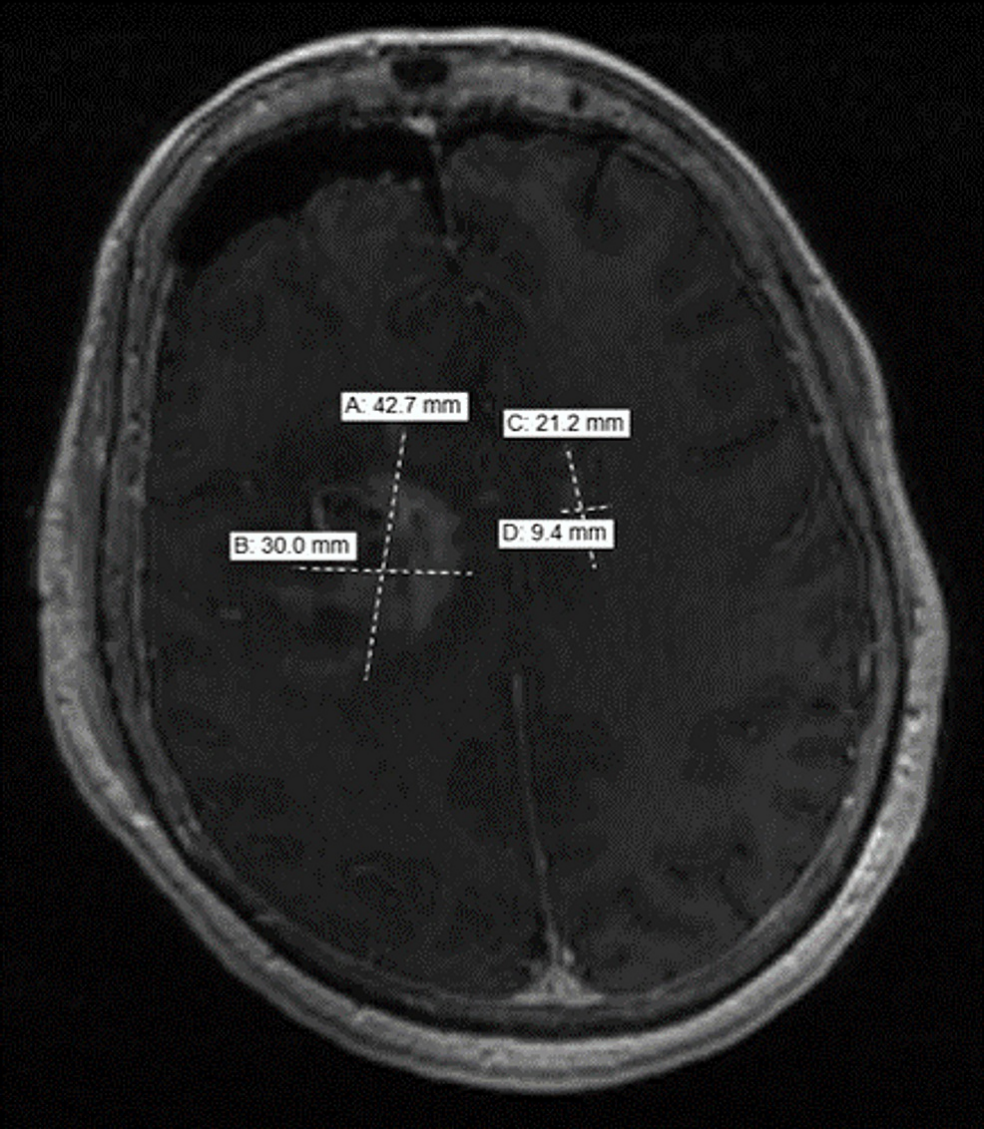
Post-op MRI showing debulked tumor with persistent surrounding edema.

A follow-up MRI of the brain revealed that surgical changes of the right frontal lobe were present. There was a tract extending through the previously seen right frontal mass which had hemosiderin deposition and hemorrhagic changes surrounding the tract. It extended from the craniotomy site in the right frontal lobe to the right basal ganglia. The large right frontal lobe mass showed a slight decrease in size with the central portion debulked. However, there was still marked surrounding edema and residual enhancing brain parenchyma. In addition, previously seen abnormal edema, which extended across the midline, into the left frontal was still noted (Figure 3).

**Figure 4:**
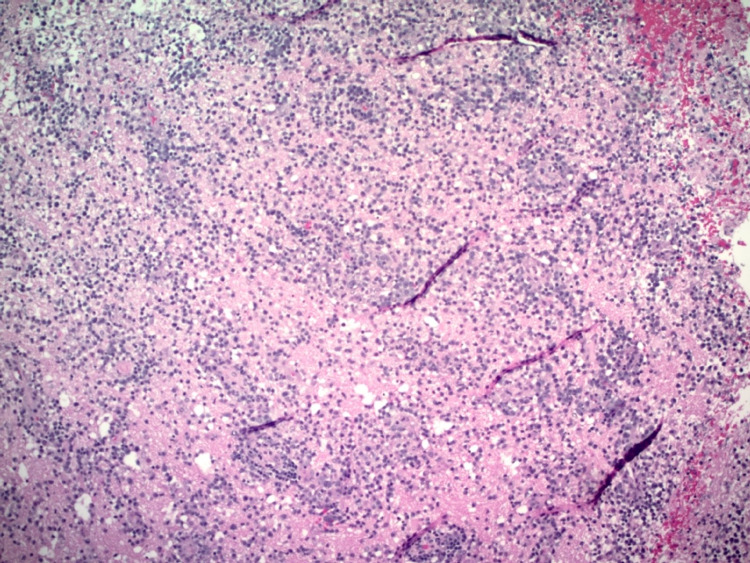
High-grade large B-cell lymphoma.

**Figure 5:**
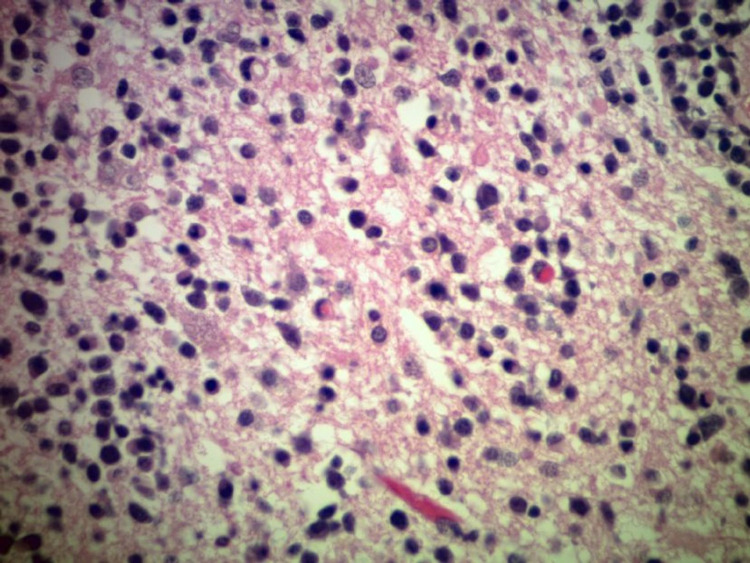
High-grade large B-cell lymphoma.

Following IV contrast administration there was still prominent enhancement surrounding the surgical changes and the residual underlying mass in the right frontal lobe, which was infiltrative in appearance and still demonstrated enhancement. It measured about 3 cm x 4.3 cm in diameter. The left-sided lesion also demonstrated enhancement, which measured about 2.1 cm x 9.4 cm. Final tissue pathology revealed high-grade large B-cell lymphoma as seen in Figures 4-5.

Immunoassay revealed atypical brain cells to be positive for CD20, PAX-5, BCL1, BCL6 (focal), MUM-1, with a high proliferation rate, estimated KI-67 >60%. The cells are negative for: CD3, CD5, CD10, CD15, CD30, CD23, BCL1, CMYC (less than 30%), CD34, TDT, and EBER. Flow cytometry analysis reported monoclonal B-cells with Lambda light chain restriction, consistent with B-cell lymphoproliferative disorder. The patient showed symptomatic improvement s/p resection and was discharged to a rehab facility. She continued on Keppra and dexamethasone with scheduled follow-up with Oncology for further management. On a follow-up visit, Oncology proposed therapy with high-dose methotrexate (MTX) with leucovorin rescue that would be administered every two weeks for 12 weeks, a total of six treatments. Prior to initiation of treatment, however, further studies were done to evaluate for metastasis including MRI and lumbar puncture (LP). LP was done to rule out cerebrospinal fluid (CSF) involvement prior to initiation of therapy and results were negative for malignant cells. MRI spine was also done which was negative for any lesions. The patient was scheduled to continue with a treatment plan as proposed and currently keeps improving symptomatically.

## Discussion

This case-based pathology description will be classified as “high-grade B-cell lymphoma with BCL6 gene arrangement.” And based on the presentation of the patient considering no systemic involvement and overall history and presentation. This aligns with an extranodal NHL involving the brain primarily, which brings us to the topic of primary central nervous system (CNS) lymphoma.

Primary central nervous system lymphoma is an uncommon variant of extranodal NHL that involves the brain, leptomeninges, eyes, or spinal cord without evidence of systemic disease. As seen in this patient, the lesion is only involving the brain without the involvement of any other organ or systemic disease. The epidemiology and clinical presentation vary depending upon the immunocompetence of the patient. PCNSL represents approximately four percent of newly diagnosed primary CNS tumors, with an age-adjusted incidence rate of four cases per million persons per year [1-2]. The incidence in the general population rose from the 1960s to the 1990s, peaked in the mid-1990s, and then declined [1, 3-13]. Such changes were largely driven by PCNSL cases in men between the ages of 20 and 64 years [14]. Thus, the trend has been attributed in large part to changes in HIV incidence and management over the same time period. By contrast, the incidence rate in adults >65 years of age has steadily risen, even in the last decade [14]. Most cases of non-HIV-related PCNSL are diagnosed in patients between 45 and 65 years of age, with a median age at diagnosis in the fifth decade [3,15-17]. Sporadic PCNSL, in apparently immunocompetent individuals, has been reported in association with other diseases. Antecedent flu-like or gastrointestinal illnesses like gastroenteritis have been seen in up to 15% of patients and many patients with PCNSL [4]. In addition, patients with autoimmune diseases such as rheumatoid arthritis, systemic lupus erythematosus, Sjögren syndrome, myasthenia gravis, sarcoidosis, and vasculitis may be predisposed to develop either disease- or therapy-related PCNSL [18]. Several distinctive radiographic features suggest the diagnosis of PCNSL in immunocompetent individuals. Approximately 50%-70% of immunocompetent patients with PCNSL develop solitary lesions [19], with the remainder (approximately 25%) developing the multifocal disease. Periventricular lesions (e.g., thalamus, basal ganglia, and corpus callosum) are most common (60%) followed by lesions in the frontal, parietal, temporal, and occipital lobes in 20%, 18%, 15%, and 4% of patients, respectively [20]. The radiographic lesion tends to be a solitary nonhemorrhagic mass, situated in the deep white matter adjacent to the ventricular surface. The borders are sharply circumscribed in the majority of lesions (87%), but maybe ill defined (15%) [20]. Although mild surrounding edema is present in the majority of cases, it is usually less profound than that which accompanies metastatic foci of carcinoma. Mass effect and tumor edema are seen in over half of the cases. Primary CNS Hodgkin’s lymphoma and low-grade PCNSL are extremely rare. The latter has a better long-term outcome than aggressive histology PCNSL. Solitary lesions of the brain concerning lymphoma can have varying presentations and management will require a thorough evaluation of risk factors.

The most common histopathologic subtype of PCNSL is diffuse large B-cell lymphoma (DLBCL). As an example, a series of 33 patients with PCNSL reported that all of these tumors were DLBCL, with the vast majority being of the centroblastic subtype [17]. The majority was positive for BCL6, MUM1, and BCL2; all were negative for CD138. Similar results were seen in a series of 51 patients with non-HIV-related PCNSL and a histologic diagnosis of DLBCL. Positivity for MUM1, BCL6, BCL2, CD10, or CD138 was seen in 84%, 61%, 49%, 18%, and 0%, respectively. High-dose MTX-based chemotherapy is a standard component of initial therapy for PCNSL. In aggregate, the available data suggest that chemotherapy regimens that include high-dose systemic MTX are more effective against PCNSL than either radiation alone or regimens that do not contain MTX. The addition of rituximab to MTX-based regimens may provide additional benefit and is well tolerated.

## Conclusions

Primary CNS lymphoma is a type of high-grade B-cell lymphoma and is a rare disease. Diagnosis can be difficult based on morphological and cytological appearance due to varying gene expression and presentation at diagnosis. It can closely mimic DLBCL which is why it was originally classified as such but it can also present as a solitary extranodal lesion as seen in primary CNS lymphoma which we see in this case. It is, therefore, essential to work up any lesion suspicious for lymphoma/glioma extensively. Multiple lab work including HIV serology, imaging with MRI to evaluate for spinal cord involvement, and LP to rule out CSF involvement needs to be done prior to initiating the treatment. Slit-lamp and ophthalmology evaluation also needs to be done to rule out eye involvement. Therapy will highly depend on tissue pathology and the extent of involvement at diagnosis. Rituximab with high-dose MTX-based chemotherapy has been proven to improve prognosis in PCNSL.
